# *Bifidobacterium lactis* combined with *Lactobacillus plantarum* inhibit glioma growth in mice through modulating PI3K/AKT pathway and gut microbiota

**DOI:** 10.3389/fmicb.2022.986837

**Published:** 2022-09-06

**Authors:** Li Wang, Sui Li, Huali Fan, Mingyu Han, Jie Xie, Junrong Du, Fu Peng

**Affiliations:** ^1^Department of Pharmacology, Key Laboratory of Drug-Targeting and Drug Delivery System of the Education Ministry, Sichuan Engineering Laboratory for Plant-Sourced Drug and Sichuan Research Center for Drug Precision Industrial Technology, West China School of Pharmacy, Sichuan University, Chengdu, China; ^2^College of Life Sciences, Sichuan Normal University, Chengdu, China

**Keywords:** glioma, gut microbiota, probiotics, PI3K/Akt signal pathway, bacterial metabolites

## Abstract

Glioma is a common primary aggressive tumor with limited clinical treatment. Recently, growing research suggests that gut microbiota is involved in tumor progression, and several probiotics can inhibit tumor growth. However, evidence for the effect of probiotics on glioma is lacking. Here, we found that *Bifidobacterium (B.) lactis* combined with *Lactobacillus (L.) plantarum* reduced tumor volume, prolonged survival time and repaired the intestinal barrier damage in an orthotopic mouse model of glioma. Experiments demonstrated that *B. lactis* combined with *L. plantarum* suppressed the PI3K/AKT pathway and down-regulated the expression of Ki-67 and N-cadherin. The glioma-inhibitory effect of probiotic combination is also related to the modulation of gut microbiota composition, which is characterized by an increase in relative abundance of *Lactobacillus* and a decrease in some potential pathogenic bacteria. Additionally, probiotic combination altered fecal metabolites represented by fatty acyls and organic oxygen compounds. Together, our results prove that *B. lactis* combined with *L. plantarum* can inhibit glioma growth by suppressing PI3K/AKT pathway and regulating gut microbiota composition and metabolites in mice, thus suggesting the potential benefits of *B. lactis* and *L. plantarum* against glioma.

## Introduction

Glioma, the most frequent primary intracranial neoplasm, represents 81% of the malignant tumors of the central nervous system. According to the 2021 World Health Organization classification, glioma is divided into four grades (grade 1 to grade 4). Unfortunately, more than 50% of the glioma patients developed glioblastoma (GBM, grade 4), which is the most malignant glioma with a mean survival time of 8 months (Ostrom et al., 2021). At present, the clinical treatment strategy of glioma is mainly maximum safe surgical resection, followed by radiotherapy and chemotherapy (temozolomide, TMZ). However, the therapy of glioma is challenged by its diffusion and invasion, which leads to high recurrence rates (Xu et al., 2020).

The complicated cross-talk between gut microbiota and tumors has been substantiated in numerous studies. Actually, polymorphic microbiomes have been identified as a new hallmark of cancer in the latest research (Hanahan, 2022). Gut dysbiosis, generally induced by oxidative stress, bacteriocins, etc., can disrupt intestinal integrity, promote bacterial translocation and induce inflammation. Inflammatory factors and bacterial metabolites can increase susceptibility to cancer and may reduce the permeability of the blood–brain barrier (BBB) and into the brain (Carlessi et al., 2021). Noteworthily, the alteration of gut microbiota composition caused by long-term antibiotic administration promoted glioma growth through regulating brain immune responses in mice (D’alessandro et al., 2020). Certain bacteria, such as *Helicobacter pylori*, *Bacteroides fragilis* and *Clostridium*, have been reported to drive tumorigenesis in the gastrointestinal tract through modulating autophagy, inflammation, oxidative stress or systemic immune responses (Matson et al., 2021). In addition, bacterial metabolites, such as short chain fatty acids (SCFA), lipopolysaccharide (LPS), serotonin, gamma-aminobutyric acid (GABA), etc., can affect brain function through stimulating the vagal nerve or passing the BBB (D’alessandro et al., 2021).

Probiotics, as naturally occurring bacteria that are extensively observed in human and animal intestine, have shown their potential against various diseases including cancer and neurodegenerative diseases. *Lactobacillus* and *Bifidobacterium* are common probiotics with positive effects in the prevention and treatment of cancers. Probiotics can repress tumor development through secreting antioxidant enzymes, regulating cell cycle associated proteins and inducing apoptosis (Mohseni et al., 2021). In the recent research, *B. bifidum* was markedly enriched in the patients who positively responded to the programmed cell death protein 1 (PD-1) blockade, and studies demonstrated that oral gavage of *B. bifidum* inhibited colon tumor growth in mice through stimulating CD8^+^ T cells (Lee et al., 2021). Recently, a study showed that gut microbiota composition was different between normal and glioma mice, the abundance of *Lactobacillus* decreased at an early stage and then gradually increased. After temozolomide treatment, *Akkermansia* and *Bifidobacterium* were enriched in glioma mice (Li et al., 2021). Fecal metabolites were also altered in glioma patients and mice, with glioma patient having a reduced levels of norepinephrine and 5-hydroxyindoleaceic acid (serotonin metabolite). Interestingly, the effects of glioma on fecal metabolites were abrogated after temozolomide treatment (Dono et al., 2020). Administration of *L. plantarum* can increase serotonin and dopamine level in the striatum of germ-free mice, and selective serotonin reuptake inhibitors can inhibit proliferation and induce apoptosis in glioma cell lines (D’alessandro et al., 2021). A study has shown that a ketogenic diet can slightly increase the survival of glioma mice through altering gut microbiota composition (McFarland et al., 2017). The supernatant of kefir, a commercial probiotic product, has dose-dependent cytotoxicity effect on U87 GBM cell line (Fatahi et al., 2021).

We aimed to investigate the effect of specific probiotic strains on glioma growth in GL261 orthotopic mouse model by gavage treatment with *B. lactis* or *L. plantarum* individually or in combination. Our previous experiments found that the combined application of *B. lactis* and *L. plantarum* was the most effective in reducing tumor volume of glioma-bearing mice. In this study, our results demonstrate that *B. lactis* combined with *L. plantarum* suppressed glioma development, at least partly by modulating phosphoinositide 3-kinase (PI3K)/AKT pathway, gut microbiota and metabolites. Collectively, these results reveal that *B. lactis* and *L. plantarum* might display potential efficacy against glioma.

## Materials and methods

### Cell culture

Cell line GL261 was bought from BeNa Culture Collection (Beijing, China) and cultured in DMEM-H/F12 media (Gibco, MA, United States) containing 10% fetal bovine serum (Gibco, MA, United States) and 1% penicillin/streptomycin (Invitrogen, MA, United States) at 37°C, 5% CO_2_ (Wang et al., 2020).

### Bacterial preparation

*Bifidobacterium lactis* (BI516) and *L. plantarum* (LP-Onlly) freeze-dried powders were purchased from Shanghai Onlly Company (Shanghai, China) and suspended in sterile saline to a concentration of 4 × 10^9^ CFU/0.2 ml (*B. lactis*: 2 × 10^9^ CFU, *L. plantarum*: 2 × 10^9^ CFU). The microbial number was counted using cell viable counting method, and bacteria was identified using molecular biological means by Guangdong Detection Center of Microbiology (Guangzhou, China). The detailed test results are shown in Supplementary Tables S1–S3, Sequence S1 and raw data is included in Data Sheet 1–4.

### Design of animal experiments

Male C57BL/6 mice (4 weeks) were purchased from Chengdu Dashuo Laboratory Animal Company (Chengdu, China) and housed at 22 ± 2°C under a 12 h: 12 h light–dark cycle in specific pathogen-free facilities. All animal experiments followed the regulations of ethics committee of the Experimental Animal Administration of Sichuan University. Two batches of C57BL/6 mice were used: one batch was used for survival curve (*n* = 7), and another batch was used for indicator detection (*n* = 11). The mice in each batch were randomly divided into three groups: sham, model (Mod) and Mod-*L. plantarum* (LP) *+ B. lactis* (BL) group. The Mod-LP + BL group was daily gavaged with 200 μl probiotic mixture of *B. lactis* (2 × 10^9^ CFU) and *L. plantarum* (2 × 10^9^ CFU) till the end of experiment. The sham and Mod group were daily gavaged with 200 μl sterile saline (Van Zyl et al., 2018). Stereotaxic surgery was performed on mice 10 days after the first gavage. The mice were intraperitoneally anesthetized with 4% chloral hydrate (66 mg/kg). Then, 1 × 10^5^ GL261 cells (in 1 μl PBS) were stereotactically injected into the right striatum in the Mod and Mod-LP + BL groups and the stereotaxic coordinates to the bregma were 1.0 mm anterior, + 1.5 mm lateral, and 3.5 mm ventral (Lam et al., 2018). The sham group was injected with 1 μl PBS. The mice used for indicator detection were sacrificed 28 days after inoculation.

### Clinical score and behavioral testing

Clinical scores and behavioral testing were daily assessed on the last 6 days before sacrifice based on previous research (Helgers et al., 2020). Detailed clinical scoring criterion is included in Supplementary Table S4. Open-field test: Locomotor activity was measured in an open-field apparatus (40 cm × 40 cm × 40 cm). The mouse was placed in the corner of the box and the spontaneous activity was recorded for 5 min using a video camera located above the box. The total motion distance and number of line crossings were assessed by EthoVision XT software (Zheng et al., 2016). Beam balance test: Motor coordination and balance were assessed by beam balance test and scoring criteria was as previously described (Supplementary Table S5; Chen et al., 2001).

### Magnetic resonance imaging

*In vivo* cerebral magnetic resonance imaging (MRI) was performed on a small animal 7 T MRI scanner (Time Medical, Shanghai, China). All mice were anesthetized with 2% inhaled isoflurane. Images were collected using a 2D T2-weighted sequence (axial orientation): Repetition time = 3,500 ms, echo time = 54 ms, number of averages = 4, matrix = 256 × 256, 6 slices, slice thickness = 1 mm. Tumor volumes were calculated using ITK-SNAP program (Agliardi et al., 2021).

### Fecal samples and tissue collection

Fresh fecal pellets were collected and immediately frozen at −80°C on days 25 to 27 after inoculation. All mice were anesthetized with 4% chloral hydrate (66 mg/kg) and intracardially perfused with cold sterilized saline at the end of experiments. Brain, ileum and colon tissues were collected, part tissues were snap-frozen in liquid nitrogen and stored at −80°C, and part tissues were fixed with 4% paraformaldehyde and embedded in paraffin (Yang et al., 2020).

### Histological and immunohistochemical analyses

Paraffin-embedded tissues were cut into 3-μm-thick slides. Hematoxylin and eosin (HE) staining was conducted according to standard histological protocols. For immunohistochemistry (IHC), slides were, respectively, incubated with the following primary antibodies from Abcam (Cambridge, United Kingdom) after blocking: Ki-67 (ab16667), Occludin (ab216327), ZO-1 (ab221547). Quantification of IHC was performed by counting immunostaining-positive cells (Ki-67) and calculating positive areas (Occludin, ZO-1) using Image Pro Plus software (Chen et al., 2020).

### Reverse transcription quantitative real-time PCR

Total RNA was isolated using TRIzol reagent (Thermo fisher scientific, MA, United States) and reverse-transcribed with the RevertAid First Strand cDNA Synthesis Kit (Thermo fisher scientific, MA, United States). Real-time-polymerase chain reaction was conducted according to previous research and data was processed by 2^−ΔΔCT^ method (Yang et al., 2020). The primer sequences for target genes are listed in Supplementary Table S6.

### Western blot

Total proteins were extracted with RIPA buffer (Beyotime, Shanghai, China) following the manufacturer’s instructions. The protein lysate was electrophoresed and transferred to a PVDF membrane (Bio-Rad, CA, United States). The membrane was, respectively, incubated with the following primary antibodies from CST (Shanghai, China): p-PI3K (Tyr458/Tyr199; 1:1000; 4,228), PI3K (1:1000; 4,257), p-AKT (Ser473; 1:2000; 4,060), AKT (1:1000; 4,691), PTEN (1:1000; 9,559), survivin (1:1000; 2,808), FoxO1 (1:1000; 2,880), Bad (1:1000; 9,292), E-Cadherin (1:1000; 3,195) and N-Cadherin (1:1000; 13,116). The proteins were detected with ECL Plus Reagent (Beyotime, Shanghai, China) and quantified using ImageJ software (Yang et al., 2020).

### Gut microbial analysis

Fecal Genomic DNA was Extracted with a Fecal DNA Extraction kit (TIANGEN, Beijing, China) Following the manufacturer’s Instructions. Amplification of the Near-Full-Length 16S Rrna Genes was Performed With the 27F/1390R Primers. Next, the Constructed Library was Sequenced on the Pacbio Sequel Platform. The High-Quality Reads Were Analyzed With QIIME Software (Stearns et al., 2011). The Qualified Sequences Were Clustered Into Operational Taxonomic Units (OTUs) at 97% Similarity. Raw Data for the 16S Rrna Sequences Were Submitted to the Genbank Databases Under Accession Number PRJNA863912.

### Next-generation sequencing

RNA-seq was performed based on previous research (Wang et al., 2019). Briefly, mRNA was purified from total RNA and used to construct RNA-seq library with NEBNext^®^ Ultra™ RNA Library Prep Kit (NEB, MA, United States). Paired-end mRNA sequencing was carried out on Noves6000 platform. Clean reads were mapped to the mouse reference genome (GRCm38.p6) using HISAT2. Differentially expressed genes (DEGs) were selected with|fold change (FC)| > 1.5 and value of *p* < 0.05 using edgeR. Gene ontology (GO) and kyoto encyclopedia of genes and genomes (KEGG) analysis were performed using the clusterProfiler package. The RNA-seq raw data have been deposited in GenBank database with the accession number of PRJNA863751.

### Untargeted metabolomics by GC–MS

Metabolic extracts from fecal were analyzed by gas chromatography–mass spectrometry (GC–MS) based on previous research (He et al., 2019). Fecal samples were handled with extract solvent (methanol-chloroform, 3:1, containing internal standard). The quality control samples were prepared by mixing an equal aliquot of all collected samples. GC–MS analyses were performed on Agilent 7,890 system with DB-5MS capillary column (30 m × 250 μm × 0.25 μm). Samples (1 μl) were injected in splitless mode. The injector, transfer line and ion source temperatures were 280°C, 280°C and 250°C, respectively. The oven temperature program was 50°C for 1 min, then raised to 310°C at 10°C/min, and held steady for 8 min. Raw GC–MS data were processed with ChromaTOF software (LECO). The raw data for GC–MS have been submitted to the Metabolights database under accession number MTBLS5563.

### Statistics analysis

Statistical analyses were carried out with the SPSS 25.0 software. Log-rank test was used for Kaplan–Meier survival curves. Statistical difference between 2 groups was identified using a two-tailed Student’s *t*-test. For multiple comparisons, two-way *ANOVA* followed by LSD *post hoc test* was performed. Differences at *p* < 0.05 were considered significant.

## Results

### *Bifidobacterium lactis* combined with *Lactobacillus plantarum* inhibit glioma growth in mice

**Figure 1:**
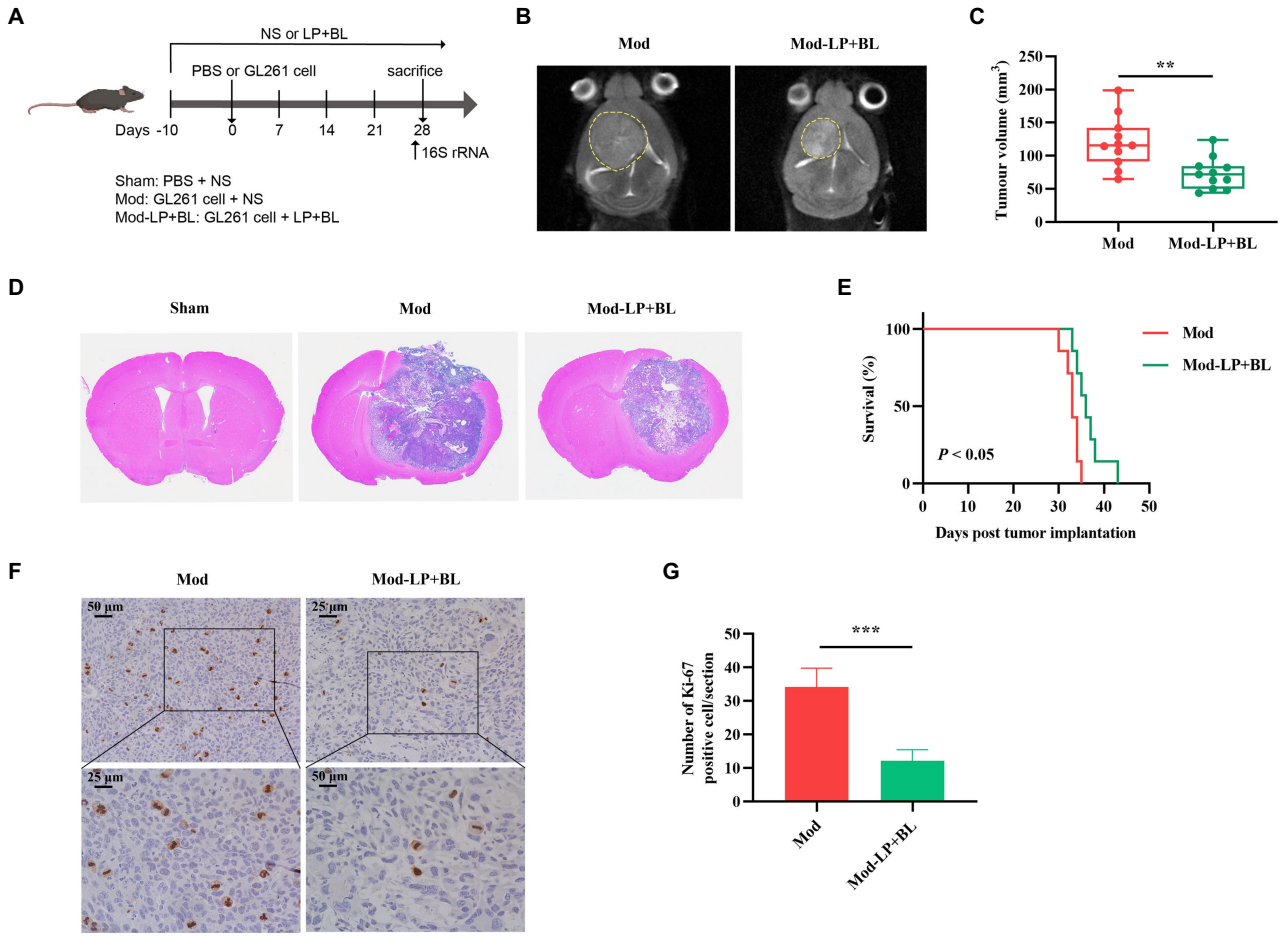
*Bifidobacterium lactis* and *Lactobacillus plantarum* inhibit glioma growth in mice. **(A)** Experimental design. **(B)** Representative T2-weighted MRI images and **(C)** tumor volume quantification of glioma mice receiving either PBS or probiotic combination at day 28 post tumor implantation, *n* = 11, two-tailed Student’s *t*-test. **(D)** Representative HE-stained coronal brain sections. **(E)** Kaplan–Meier survival curves, *n* = 7, Log-rank test. **(F)** Representative Ki-67-stained sections and **(G)** numbers of Ki-67 positive cells of brain tissues, scale bars, 50 μm (upper) or 25 μm (lower). *n* = 6. Two-tailed Student’s *t*-test. Mod, Model. LP, *Lactobacillus plantarum*; BL, *Bifidobacterium lactis*; NS, normal saline. ^*^p < 0.05, ^**^p < 0.01, ^***^p < 0.001.

To determine whether *B. lactis* combined with *L. plantarum* can inhibit glioma growth in mice, we treated glioma-bearing mice with *B. lactis* and *L. plantarum via* daily oral gavage (Figure 1A). Tumor volume of glioma-bearing mice was evaluated by cerebral T2-weighted MRI sequence on day 28 post-inoculation (Figure 1B). Quantitative analysis showed that compared with the Mod group, tumor volume of the Mod-LP + BL group was declined (120.36 ± 38.69 mm^3^ vs. 73.25 ± 23.88 mm^3^, *p* < 0.01; Figure 1C). HE staining of glioma tissues substantiated this point (Figure 1D). As shown in Figure 1E, *B. lactis* combined with *L. plantarum* slightly prolonged the median survival time of glioma mice (33.0 days vs. 37.0 days, *p* < 0.05). To examine whether probiotics regulate tumor proliferation, IHC staining for Ki-67 was performed, and the number of Ki-67 positive cells was decreased in the Mod-LP + BL group compared to the Mod group (Figures 1F,G).

### Effects of *Bifidobacterium lactis* and *Lactobacillus plantarum* on neurobehavior in glioma mice

**Figure 2:**
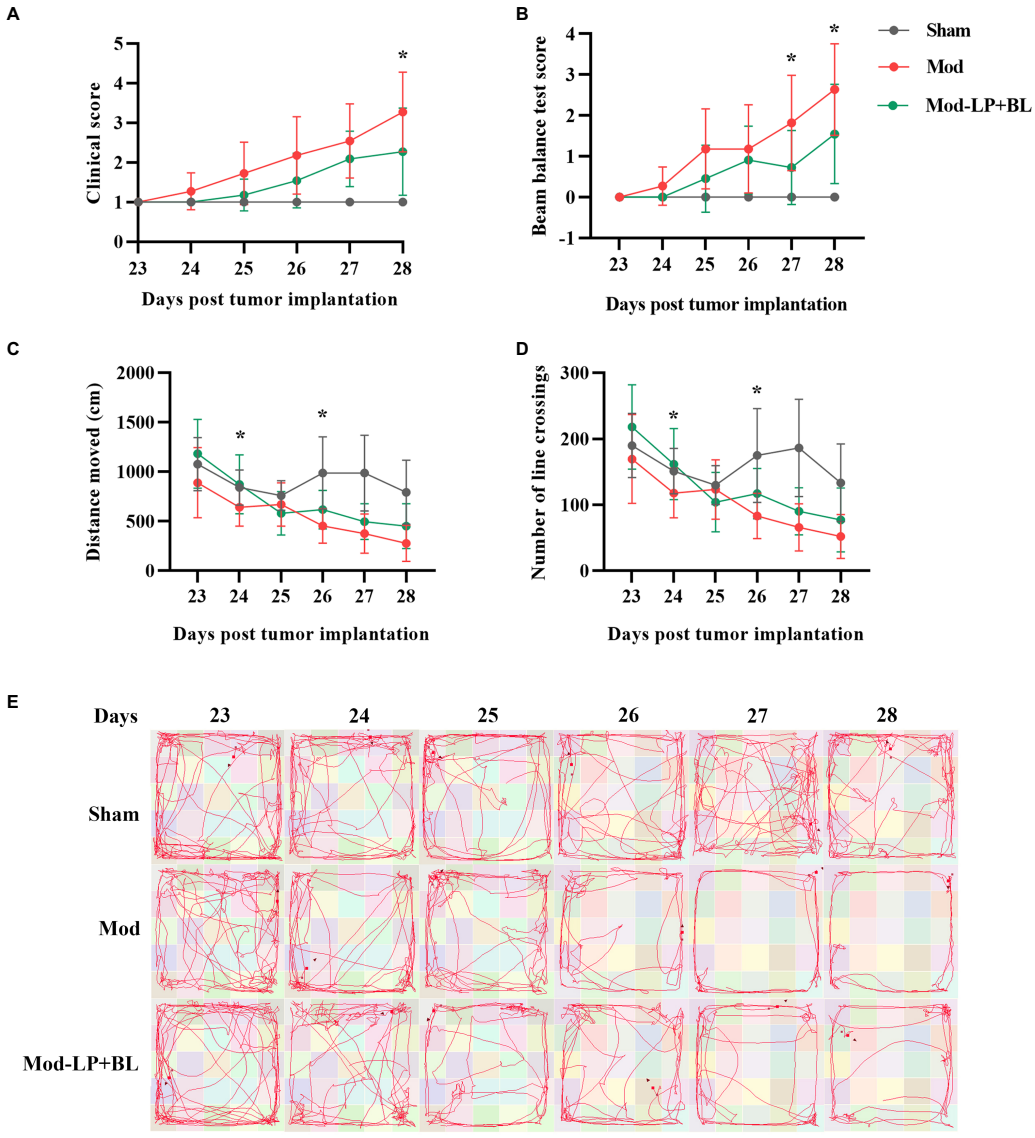
Effects of *B. lactis* and *L. plantarum* on neurobehavior in glioma mice. **(A)** Clinical score and **(B)** beam balance test score of mice at day 23 to 28 post tumor implantation. **(C)** Distance moved and **(D)** number of line crossings in the open field of mice at day 23 to 28 post tumor implantation. **(E)** Representative images of the movement track of mice in the open-field test. Mod, Model. LP, *Lactobacillus plantarum*; BL, *Bifidobacterium lactis*. Two-way *ANOVA*. ^*^p < 0.05. *n* = 11.

To explore the influences of *B. lactis* and *L. plantarum* on neurobehavior in glioma mice, we performed clinical scoring and behavioral testing. With the tumor growth, the locomotor ability and motor coordination of the mice in the Mod group deteriorated rapidly on the 23rd to 28th day after inoculation compared with the sham group, and probiotic mix alleviated this symptom. Compared with the Mod group, the clinical score and beam balance test score of the Mod-LP + BL group were decreased (day 27 and day 28: *p* < 0.05; Figures 2A,B). Open-field test demonstrated that distance moved and number of line crossings of the Mod group continuously declined compared with the sham group, and probiotic mix slowed down this trend (day 24 and day 26: *p* < 0.05; Figures 2C–E).

### *Bifidobacterium lactis* combined with *Lactobacillus plantarum* repair intestinal barrier damage in glioma mice

**Figure 3:**
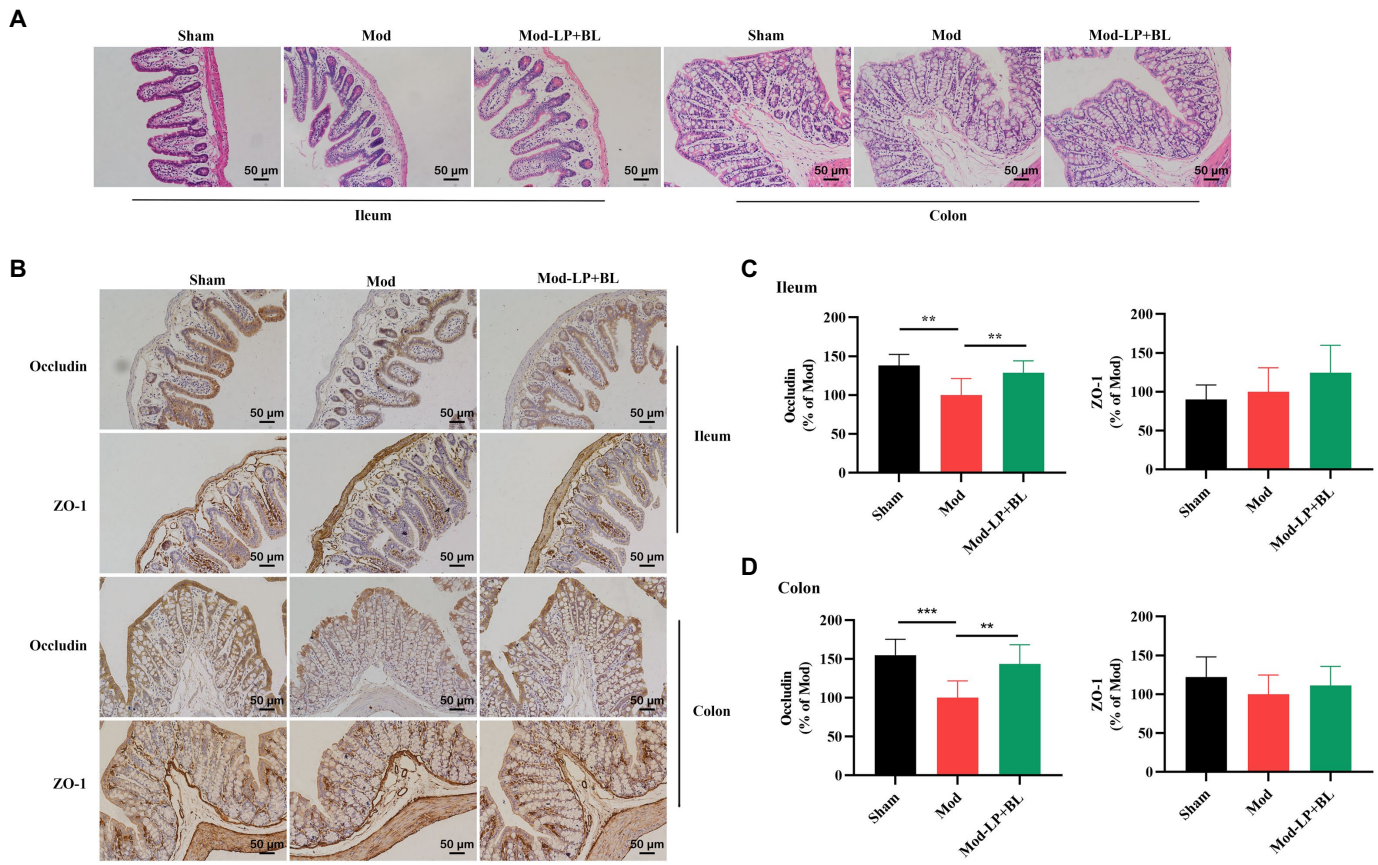
*B. lactis* and *L. plantarum* repair intestinal barrier damage in glioma mice. Representative **(A)** H&E, **(B)** ZO-1 and Occludin stained sections of ileum and colon tissues harvested from mice, scale bars, 50 μm. Quantification of ZO-1 and Occludin immunopositive area in **(C)** ileum and **(D)** colon tissues, two-way *ANOVA*. Mod, Model. LP, *Lactobacillus plantarum*; BL, *Bifidobacterium lactis*. ^*^p < 0.05, ^**^p < 0.01, ^***^p < 0.001. *n* = 6.

The intestinal defense system plays a pivotal role in preventing the bacterial overgrowth and mucosal immune response. Intercellular tight junctions, which are constituted by Occludin, claudin, zonula occludens (ZO) and junctional adhesion, are the major determinants of the intestinal physical barrier (Suzuki, 2020). HE staining did not show obvious abnormality in ileum and colon tissues of mice in each group (Figure 3A). To explore the effect of probiotics on intestinal barrier in glioma mice, we detected the expression of tight junction proteins. IHC staining showed that protein expression of Occludin in ileum and colon tissues was down-regulated in the Mod group compared with the Sham group (Ileum, *p* < 0.01; colon, *p* < 0.001), and probiotic combination up-regulated Occludin expression in ileum and colon tissues (Ileum, *p* < 0.01; colon, *p* < 0.01; Figures 3B–D).

### *Bifidobacterium lactis* combined with *Lactobacillus plantarum* inhibit PI3K/AKT pathway in glioma mice

**Figure 4:**
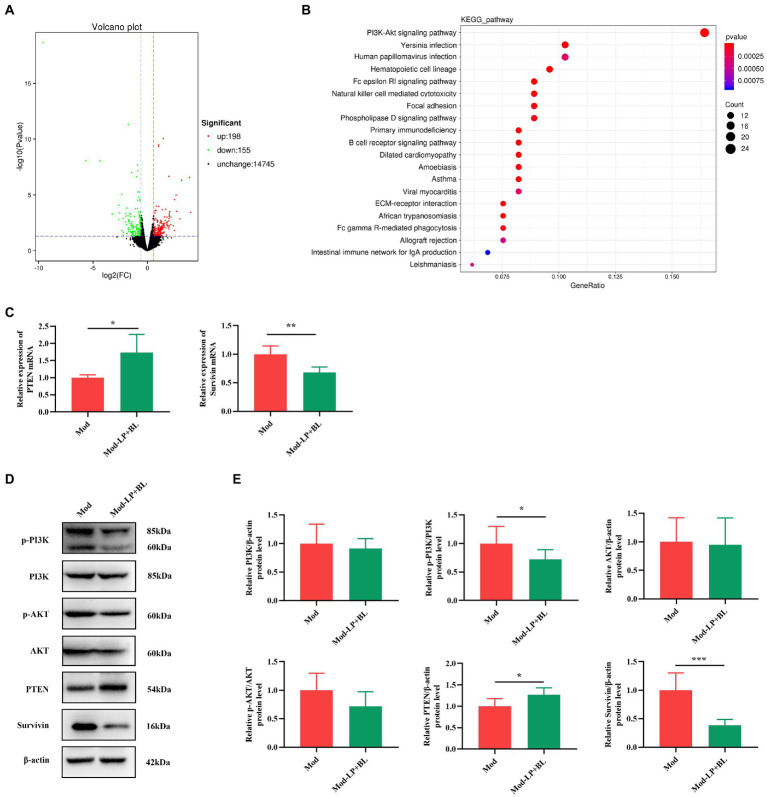
*B. lactis* combined with *L. plantarum* inhibit PI3K/AKT pathway in glioma mice. **(A)** Volcano plot of DEGs of glioma tissues between the Mod and Mod-LP + BL groups. *n* = 3. **(B)** KEGG pathway enrichment analysis of DEGs. **(C)** The mRNA expression of *PTEN* and *survivin* in glioma tissues. *n* = 6. Two-tailed Student’s T-test. **(D)** Representative immunoblots and **(E)** quantitative analysis of p-PI3K, p-AKT, PTEN and survivin in glioma tissues. *n* = 6. Two-tailed Student’s T-test. Mod, Model. LP, *Lactobacillus plantarum*; BL, *Bifidobacterium lactis*. ^*^p < 0.05, ^**^p < 0.01, ^***^p < 0.001.

To delve the potential mechanism of the inhibitory effect of probiotics on glioma, RNA-seq was performed on the glioma tissues of mice. DEGs between the Mod and Mod-LP + BL groups were identified based on |FC| > 1.5 and value of *p* <0.05, among which 198 were up-regulated and 155 were down-regulated (Figure 4A). Then, a transcriptional heatmap was constructed to compare the expression profiles of DEGs between different groups, and the comparison showed significantly transcriptomic alterations in the Mod-LP + BL group compared with the Mod group (Supplementary Figure S1). GO analysis of DEGs revealed enrichment in cell adhesion, extracellular space and calcium ion binding (Supplementary Figure S2). KEGG pathway enrichment analysis predicted that PI3K-AKT signaling pathway involved in the regulation of probiotics on glioma (Figure 4B).

PI3K/AKT pathway, as the most enriched pathway identified by KEGG analysis, plays a crucial role in tumor initiation and progression. As a negative regulator of PI3K signals, the loss of phosphatase and tensin homolog deleted on chromosome 10 (PTEN) results in PI3K pathway activation (Lee et al., 2018). Abnormal activation of PI3K and its downstream signals (Bad (Bui et al., 2018), FoxO1 (Yan and Huang, 2019), survivin (Yang et al., 2017), etc.) can promote carcinogenesis. To assess the activation of PI3K/AKT pathway, q-PCR and western blot of glioma tissues were performed. As shown in Figure 4C, probiotic combination up-regulated the mRNA expression of *PTEN* and down-regulated the mRNA expression of *survivin* in glioma tissues (*PTEN*, *p* < 0.05; *survivin*, *p* < 0.01; Figure 4C). At the protein level, probiotic combination down-regulated p-PI3K and survivin (p-PI3K, *p* < 0.01; survivin, *p* < 0.001; Figures 4D,E), and up-regulated PTEN expression in glioma tissues (*p* < 0.05; Figures 4D,E). Nevertheless, the protein expression of Bad and FoxO1 did not change significantly (Supplementary Figure S3).

### Effects of *Bifidobacterium lactis* and *Lactobacillus plantarum* on the migration and invasion-related genes in glioma mice

**Figure 5:**
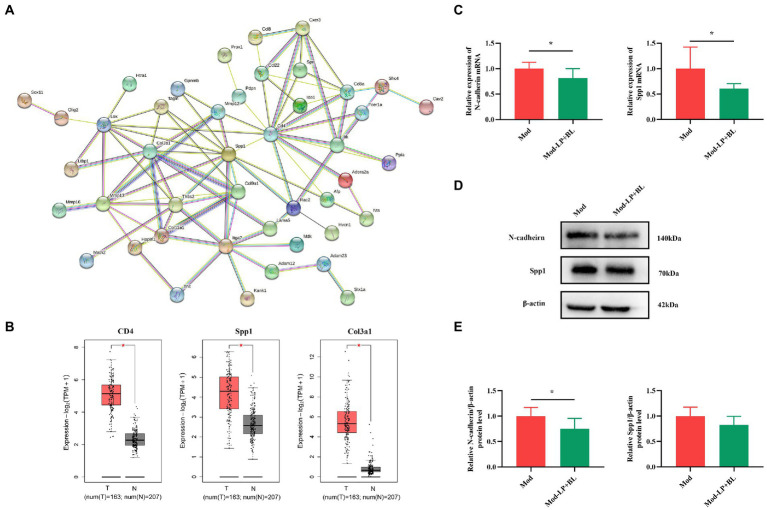
Effects of *B. lactis* and *L. plantarum* on the migration and invasion-related genes in glioma mice. **(A)** PPI network. **(B)** Differential expression of *CD4*, *Spp1* and *Col3a1* in normal and glioma tissues from TCGA and GTEx datasets. **(C)** mRNA expression of *N-cadherin* and *Spp1* in glioma tissues in Mod and Mod-LP + BL groups. n = 6, two-tailed Student’s T-test. **(D)** Representative immunoblots and **(E)** quantitative analysis of N-cadherin and Spp1 in glioma tissues. *n* = 6, two-tailed Student’s T-test. Mod, Model. LP, *Lactobacillus plantarum*; BL, *Bifidobacterium lactis*. T, tumor. N, normal. ^*^p < 0.05, ^**^p < 0.01, ^***^p < 0.001.

To predict interactions between candidate genes at the protein level, candidate genes were selected by taking the intersection of DEGs and glioma-associated genes, and a protein–protein interaction (PPI) network was constructed using the STRING database. The PPI network consisted of 44 nodes and 89 edges, and *Cd4*, *Spp1* and *Col3a1* were identified as the top three hub genes (Figure 5A). Subsequently, the cancer genome atlas (TCGA) and the genotype-tissue expression (GTEx) datasets were analyzed by GEPIA to evaluate the differential expression and prognostic value of the three hub genes. Compared with normal tissues, *Cd4*, *Spp1* and *Col3a1* expression were significantly increased in glioma tissues (*Cd4*, *p* < 0.05; *Spp1*, *p* < 0.05; *Col3a1*, *p* < 0.05; Figure 5B), but their expression levels were not correlated with overall survival (Supplementary Figure S4).

PPI network showed that genes related to tumor migration and invasion were different in the Mod and Mod-LP + BL groups. Osteopontin (OPN/Spp1), a potential biomarker for glioblastoma, could induce glioma cell migration and invasion. Experiments demonstrated that selective knockdown of osteopontin displayed an anti-tumoral activity in a GBM model (Lamour et al., 2010). Tumor invasion and metastasis are generally associated with epithelial-mesenchymal transition, which is a cellular process accompanied with the loss of epithelial marker (E-cadherin, etc.) and increase in mesenchymal markers (N-cadherin, vimentin, etc.) (Pastushenko and Blanpain, 2019). q-PCR was conducted to prove that mRNA expression of *Spp1* and *CDH2/*N-cadherin are down-regulated by probiotic combination in glioma mice (*Spp1*, *p* < 0.05; N-cadherin, *p* < 0.05; Figure 5C). Western blot showed that probiotic combination suppressed N-cadherin protein expression (*p* < 0.05), but did not statistically affect the protein expression of Spp1 (Figures 5D,E) in glioma tissues. Additionally, the mRNA and protein expression of *CDH1/*E-cadherin did not alter in the Mod-LP + BL group compared with the Mod group (Supplementary Figure S5). The original images of western blot are shown in Supplementary Figure S6.

### *Bifidobacterium lactis* combined with *Lactobacillus plantarum* altered the gut microbiota composition in glioma mice

**Figure 6:**
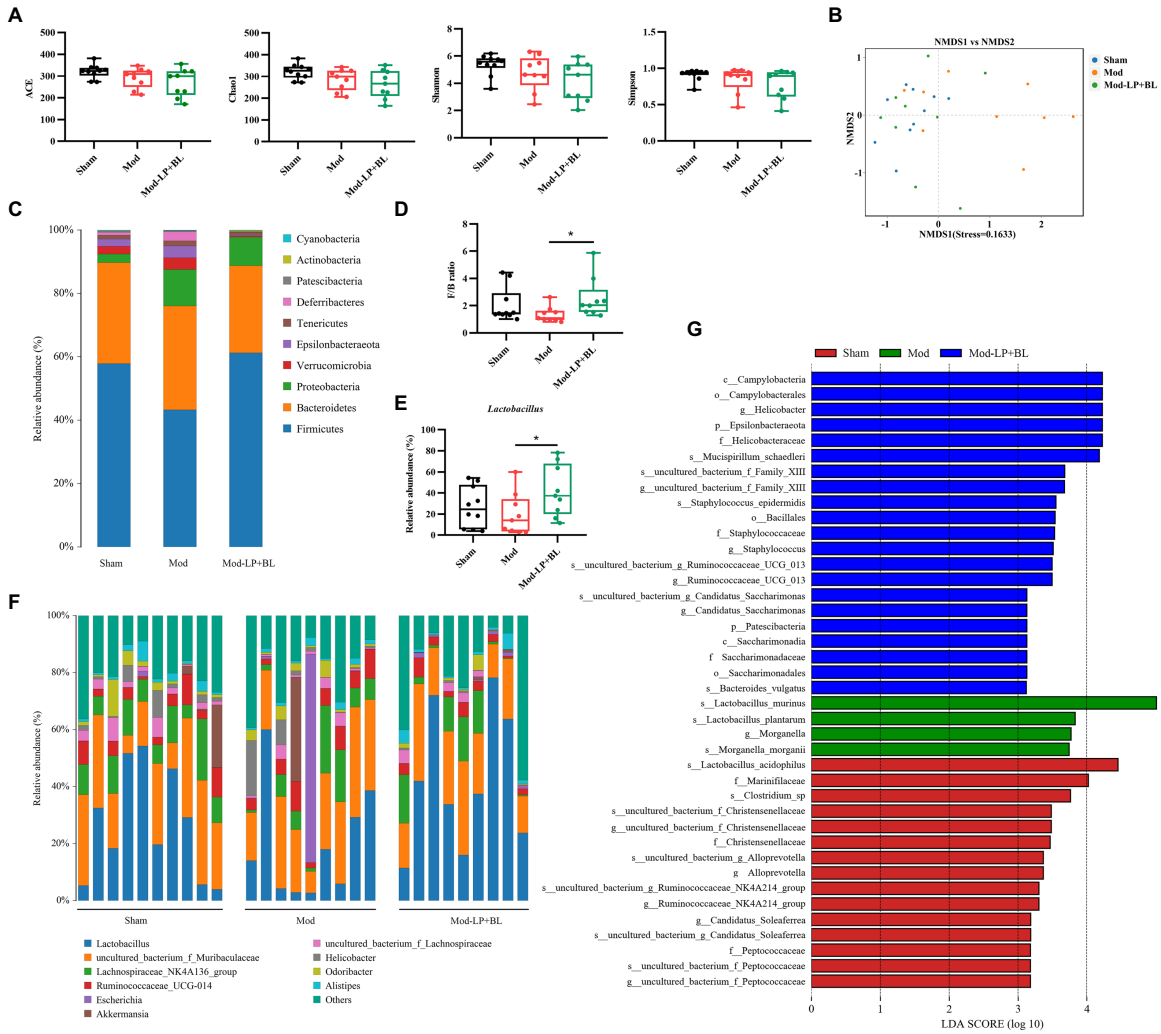
*B. lactis* combined with *L. plantarum* altered the gut microbiota composition in glioma mice. **(A)** Comparison of α-diversity of gut microbiota including ACE, Chao1, Simpson and Shannon index between the three groups. Wilcoxon rank-sum test. **(B)** NMDS analysis based on binary-jaccard distance, stress = 0.1633. Bar charts of the gut microbiota composition at the **(C)** phylum and **(F)** genus-level in the three groups. **(D)** F/B ratio, two-way *ANOVA*. **(E)** Relative abundance of *Lactobacillus* in the three groups, two-way *ANOVA*. **(G)** LDA score of enriched bacterial taxa, LDA > 3.0. Mod, Model. LDA, Linear discriminant analysis. LP, *Lactobacillus plantarum*; BL, *Bifidobacterium lactis*. ^*^p < 0.05. *n* = 9–10.

To compare the alteration of the gut microbiota composition in the three treatment groups, 16S rRNA sequencing was conducted on feces from mice. The α-diversity of gut microbiota was analyzed by four metrics: ACE, Chao1, Simpson and Shannon index, and results showed that *B. lactis* combined with *L. plantarum* did not affect the α-diversity of gut microbiota in glioma mice (Figure 6A). For β diversity, the nonmetric multidimensional scaling (NMDS), based on binary-jaccard distance, exposed insignificant clustering of gut microbiota composition between glioma and normal mice, but probiotic combination conspicuously affect the composition of gut microbiota in glioma mice (Figure 6B). As shown in Figure 6C, gut microbiota in the three groups was dominated by *Bacteroidetes* and *Firmicutes* at the phylum level. A lower abundance of *Firmicutes* was observed in the Mod group compared with the Sham group, and probiotic combination increased the abundance of *Firmicutes* and decreased the abundance of *Bacteroidetes*, leading to a rising in the ratio of *Firmicutes*/*Bacteroidetes* (F/B) compared with the Mod group (2.54 ± 1.48 vs. 1.35 ± 0.58, *p* < 0.05; Figure 6D). Genus-level analysis exhibited that *Lactobacillus*, the predominant bacterium in the three groups, was reduced in glioma mice compared with healthy mice, whereas oral gavage of probiotics increases the relative abundance of *Lactobacillus* (42.04% ± 24.26% vs. 19.52% ± 19.71%, *p* < 0.05; Figures 6E,F). To further identify the specific microbiota, linear discriminant analysis effect size (LEfSe) was performed and results showed that numerous pathogenic bacteria were enriched in the Mod group, including *Helicobacter* and *Staphylococcus* (Figure 6G).

### Effects of *Bifidobacterium lactis* and *Lactobacillus plantarum* on microbial metabolites in glioma mice

**Figure 7:**
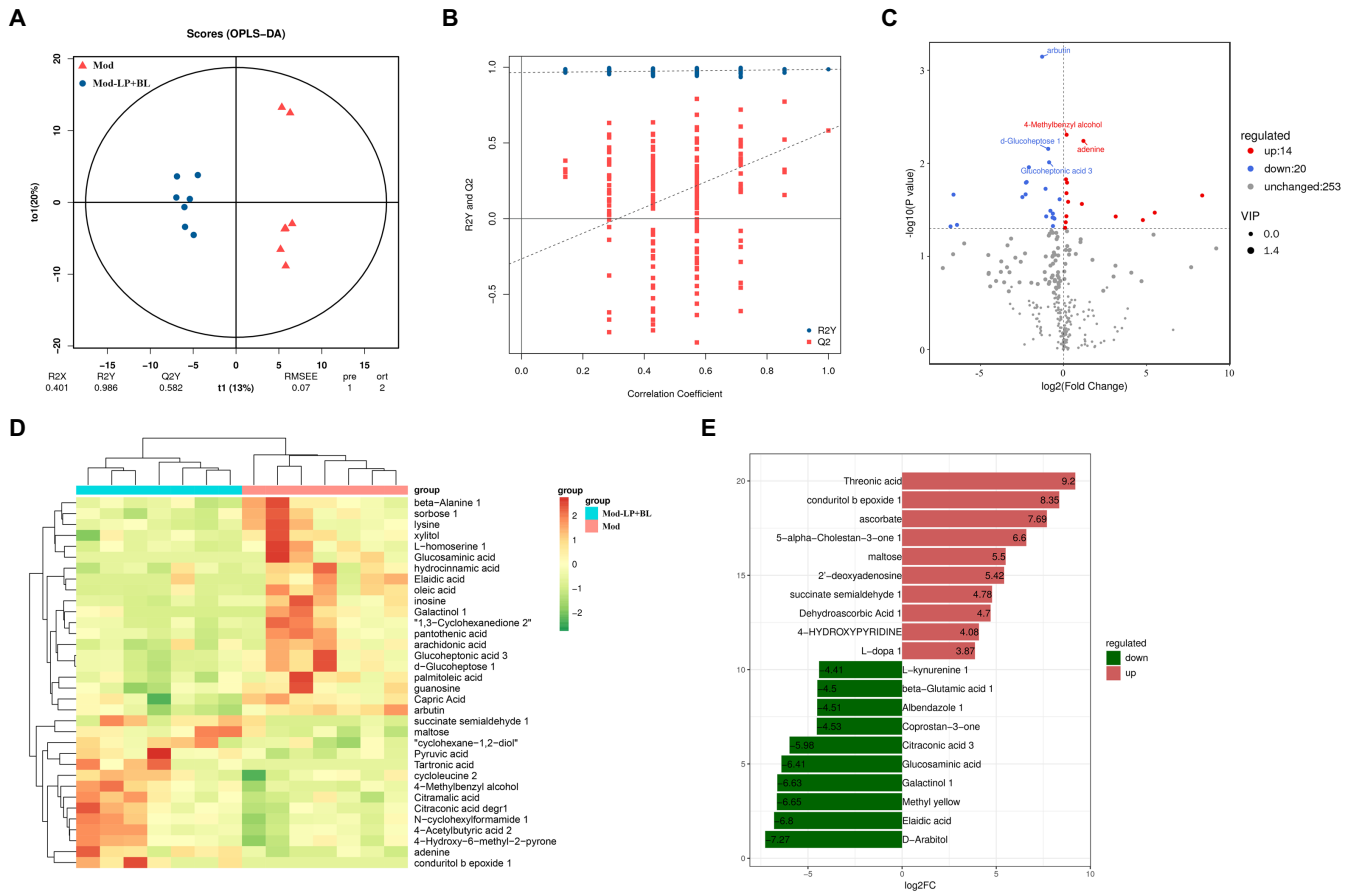
Effects of *B. lactis* and *L. plantarum* on microbial metabolites in glioma mice. **(A)** OPLS-DA score plot and **(B)** permutation tests of OPLS-DA models. **(C)** Differential fecal metabolites volcano plot of the Mod and Mod-LP + BL groups. **(D)** Unsupervised hierarchical heatmap of differential fecal metabolites between the Mod and Mod-LP + BL groups. **(E)** Fold change of differential metabolites.

Untargeted metabolomics by GC–MS was conducted on stool collected from mice in the Mod and Mod-LP + BL groups. Supervised orthogonal projections to latent structures discriminant analysis (OPLS-DA) was performed to compare the difference between the two groups. The representative OPLS-DA score plot indicated distinct separation between the Mod and Mod-LP + BL groups (R2Y = 0.986; Q2Y = 0.582; Figure 7A), and permutation tests further validated the constructed OPLS-DA model (Figure 7B). In addition, variable importance in projection (VIP) score was calculated, which is a measure of estimating the importance of each metabolite in the OPLS-DA model. With VIP > 1 and *p* < 0.05, 14 up-regulated metabolites and 20 down-regulated metabolites were identified from feces of the Mod and Mod-LP + BL groups (Figure 7C). Unsupervised analysis of all differential metabolites clearly discriminated two clusters, one gathered glioma mice treated with probiotics and another gathered glioma mice treated with PBS (Figure 7D). The primary differences were the markedly increased/decreased level of threonic acid (FC = 9.2), conduritol b epoxide 1 (FC = 8.35), ascorbate (FC = 7.69), D − Arabitol (FC = −7.27) and Elaidic acid (FC = −6.8; Figure 7E). Analysis of metabolites *via* the human metabolome database showed that differential metabolites mainly enriched in fatty acyls and organooxygen compounds (Supplementary Figure S7).

### Combined analysis of metabolomics and microbiome

**Figure 8:**
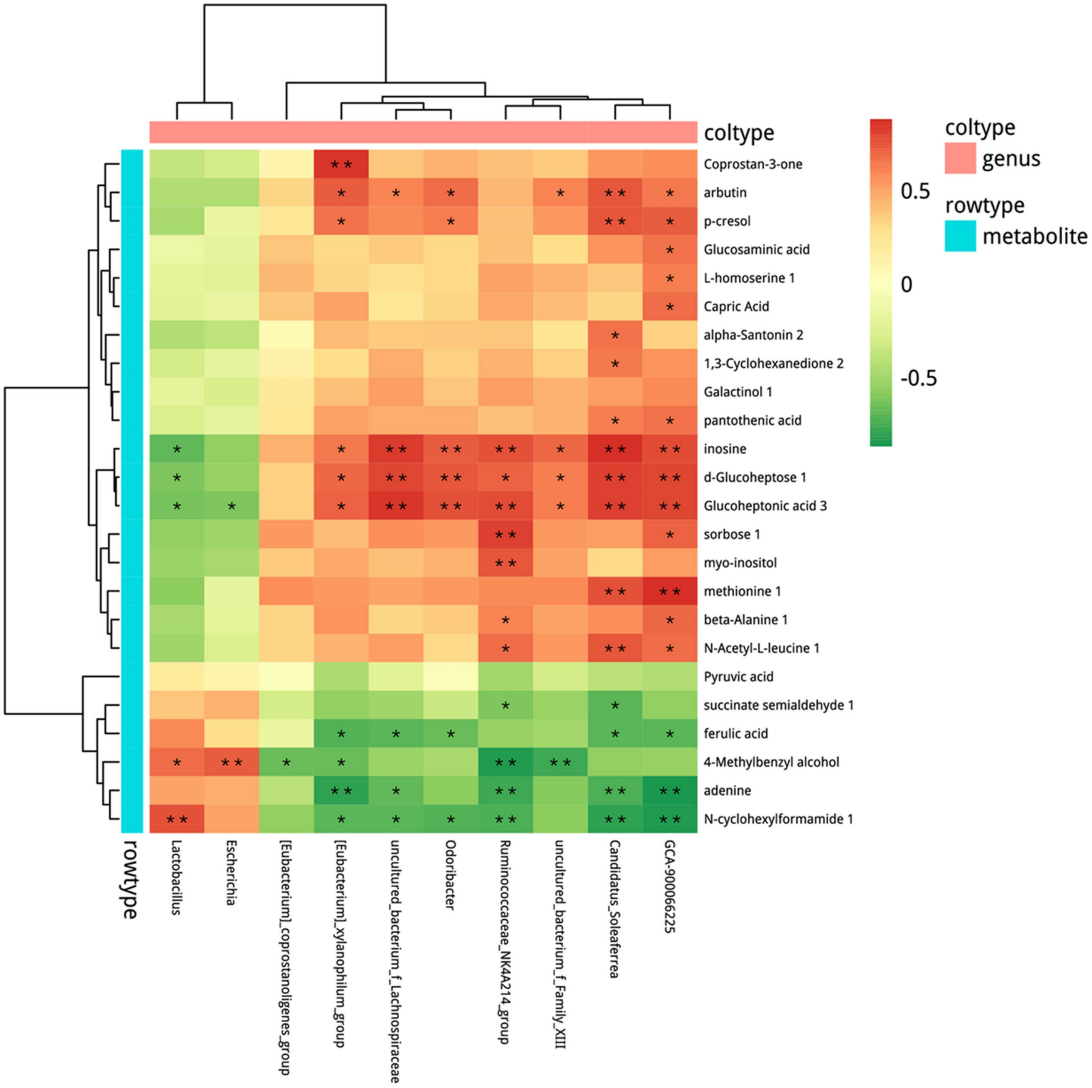
Combined analysis of metabolomics and microbiome. Spearman’s rank correlation. ^*^p < 0.05, ^**^p < 0.01. *n* = 5–6.

Based on the alteration in microbial composition and the metabolites, we combined microbiome with metabolomics to explore the possible relationship between microbes and metabolites. Correlation analysis between OTU and all differential fecal metabolites was conducted (Supplementary Figure S8) and the results are shown in Figure 8 with *p* < 0.05 at the genus level. Microbes such as *Lactobacillus*, were significantly increased after probiotics treatment and showed a positive correlation with N-cyclohexylformamide 1, 4-methylbenzyl alcohol, and a negative correlation with glucoheptonic acid 3, d-glucoheptose 1, inosine.

## Discussion

Recently, numerous researches have focused on the cross-interaction between gut microbiota and glioma (D’alessandro et al., 2021). Diverse studies have shown the distinct separation of gut microbiota between normal and glioma individuals both in humans and mice (Patrizz et al., 2020; Li et al., 2021). Notably, gut dysbiosis, caused by antibiotic supplements, can promote tumor growth in glioma mice *via* affecting immune system (D’alessandro et al., 2020; Fan et al., 2022). Therefore, we treated glioma mice with probiotics to determine whether probiotics can repress glioma progression. Our experiments show that *B. lactis* combined with *L. plantarum* can suppress glioma growth through regulating PI3K/AKT pathway and gut microbiota composition in mice. Additionally, the inhibitory effect was also associated with the alteration of fecal metabolites. Our results indicate the potential protective effects of *B. lactis* and *L. plantarum* against glioma.

The PI3K/AKT signaling pathway plays a crucial role in regulating cellular functions comprising cell survival, proliferation, growth and metabolism. Thorough molecular studies have identified RTK/RAS/PI3K (88%) as one of the significantly altered pathways in human GBMs (Colardo et al., 2021). Epidermal growth factor receptor (EGFR), a type of receptor tyrosine kinases, can recruit and active PI3K/AKT pathway. More than 50% GBMs have EGFR amplifications or mutations (Oprita et al., 2021), and PIK3CA mutation (15%) and PTEN deletion can also active PI3K/AKT pathway (Colardo et al., 2021). Actually, accumulating evidence has demonstrated that probiotics, including *B. lactis* and *L. plantarum*, can down-regulate hyperactivated PI3K/AKT pathway in numerous diseases both *in vivo* and *in vitro*, such as colorectal cancer, gastric cancer and aging (Mohseni et al., 2021). In the present study, *L. plantarum* induced apoptosis in a gastric cancer cell line (AGS) through up-regulating PTEN, Bax, TLR4 and down-regulating Akt (Maleki-Kakelar et al., 2020). Experiments showed that *B. animalis* subsp. *lactis* can prevent the *Salmonella* infection through suppressing PI3K/Akt signal pathway (Huang and Huang, 2016). Our study shows that *B. lactis* combined with *L. plantarum* down-regulated the protein expression of p-PI3K, down-regulated the protein and mRNA expression of survivin, and up-regulated the protein and mRNA expression of PTEN in glioma mice. Noteworthily, *Helicobacter* (*H.*) *pylori* infection can induce reactive oxygen species- (ROS-) mediated DNA damage and activate PI3K/Akt pathway in gastric cancer both *in vivo* and *in vitro* (Xie et al., 2018). After infection with *Staphylococcus aureus*, the mammary glands of mice showed high levels of PI3K, AKT, and p-NF-κB (Han et al., 2020). Researches have depicted that staphylococcal superantigen can induce toxicity by activating PI3K/Akt pathway (Krakauer et al., 2010; Krakauer, 2012). Our results show that *B. lactis* combined with *L. plantarum* decreased the abundance of *Staphylococcus* and *Helicobacter*, therefore, we speculated that the down-regulation of PI3K/AKT pathway may be partly associated with the decrease in *Staphylococcus* and *Helicobacter.*

A recent study showed that increased abundance of *Bacteroidetes* and decreased abundance of *Firmicutes* were observed in glioma mice compared with health mice, and the decreased abundance of *Bacteroidetes* was related to delayed glioma progression (Fan et al., 2022). Our results show an analogous trend, with probiotics reducing the abundance of *Bacteroidetes* and raising the abundance of *Firmicutes*. After oral gavage of *B. lactis* and *L. plantarum*, abundance of *L. plantarum* was increased but *B. lactis* did not colonize. Numerous studies have shown that both colonized and non-colonized probiotics can benefit the host (Xiao et al., 2003; Plaza-Diaz et al., 2019; Van Zyl et al., 2020). We speculated that the antitumor effect of *B. lactis* in glioma mice can also be exerted by allowing it to stay in the intestine for a certain period of time each day. The findings of Se-Hoon et al. validated our guesses, daily gavage of different *Bifidobacterium* strains reduced tumor burden in mice. However, these strains did not colonize, and the results showed that the abundance of *Bifidobacterium* strains increased 4 h after gavage, but decreased significantly after 24 h (Lee et al., 2021). Additionally, *B. lactis* and *L. plantarum* decreased the abundance of several potential pathogenic bacteria. Uncontrolled proliferation of pathogens can contribute to dysbiosis and further lead to decreased permeability of intestinal mucosal barrier (Iacob and Iacob, 2019). Previous researches have shown that *H. pylori* can disrupt claudin-4 and claudin-5 through activating myosin light-chain kinase. Actually, proteases of the high temperature requirement A (HtrA), which are broadly expressed by various bacteria, can cleave the junction factors such as Occludin and claudin-8 (Backert et al., 2018). In this research, protein expression of Occludin in ileum and colon tissues were down-regulated in glioma mice, which means intestinal barrier was damaged. *B. lactis* combined with *L. plantarum* repaired the intestinal barrier damage in glioma mice.

Accumulating evidence demonstrates that microbial metabolites are involved in development of various tumors. Some microbiota-derived metabolites, such as SCFA (acetate, propionate, butyrate, etc.) can suppress tumor growth, while other metabolites, such as secondary bile acids can promote tumor development (Liu et al., 2020; Mirzaei et al., 2021; Zhang et al., 2021). Interestingly, microbial metabolites can penetrate the damaged intestinal barrier, even cross the BBB into the brain, modulating brain functions (Carlessi et al., 2021). In addition, bacterial metabolites can affect the vagal nerve by stimulating enteroendocrine cells or provoking enteric nervous system, regulating brain functions (D’alessandro et al., 2021). A study demonstrated that *Lactobacillus rhamnosus* can increase GABA levels in the brain (cortical regions) and reduce depression- and anxiety-related behavior in mice, while the behavioral and neurochemical effects were not observed in vagotomized mice (Bravo et al., 2011). In our research, we performed untargeted GC–MS analysis of fecal metabolites and detected a total of 253 metabolites, including 14 up-regulated metabolites and 20 down-regulated metabolites. Numerous substances with antitumor activity such as ascorbate were identified as up-regulated metabolites, and potential carcinogen such as elaidic acid and methyl yellow were identified as down-regulated metabolites. Ascorbate is an antioxidant that scavenges ROS and prevents DNA damage, and its anticancer effects have been found in a variety of tumors (Pawlowska et al., 2019). A study showed that high dose ascorbate can cause double stranded breaks and aggregate in S-phase in GBM cell lines (Castro et al., 2017). Elaidic acid, a *trans* fatty acids, can promote metastasis of tumor cell lines by activating EGFR (Kishi et al., 2018).

Taken together, our results indicate that *B. lactis* combined with *L. plantarum* repressed glioma development at least partly by regulating PI3K/AKT pathway and the gut microbiota. Therefore, *B. lactis* and *L. plantarum* might be regarded as promising candidates for the treatment of glioma.

## Data availability statement

The datasets presented in this study can be found in online repositories. The names of the repository/repositories and accession number(s) can be found below: NCBI, PRJNA863912 (16S rRNA genes) and PRJNA863751 (RNA-seq).

## Ethics statement

The studies involving animal subjects were reviewed and approved by the ethics committee of Experimental Animal Administration of Sichuan University.

## Author contributions

LW: methodology, investigation, formal analysis and writing-original draft preparation, writing-review and editing. SL, HF, MH, and JX: methodology, supervision and writing-review and editing. JD and FP: conceptualization, project administration and funding acquisition. All authors contributed to the article and approved the submitted version.

## Funding

The study was supported by National Natural Science Foundation of China (no.82003879 and U19A2010) the Key Project of Science and Technology Department of Sichuan Province (no. 2020YFS0053; 2021YFS0044), and Youth Talent Promotion Project of China Association for Science and Technology (CACM-2020-QNRC1-01) and the Open Research Fund of Chengdu University of Traditional Chinese Medicine Key Laboratory of Systematic Research of Distinctive Chinese Medicine Resources in Southwest China.

## Conflict of interest

The authors declare that the research was conducted in the absence of any commercial or financial relationships that could be construed as a potential conflict of interest.

## Publisher’s note

All claims expressed in this article are solely those of the authors and do not necessarily represent those of their affiliated organizations, or those of the publisher, the editors and the reviewers. Any product that may be evaluated in this article, or claim that may be made by its manufacturer, is not guaranteed or endorsed by the publisher.
