# Supplementary material for: Bifidobacterium lactis combined with Lactobacillus plantarum inhibit glioma growth in mice through modulating PI3K/AKT pathway and gut microbiota
Source: Front Microbiol. 2022 Sep 6;13:986837. doi: 10.3389/fmicb.2022.986837 (PMC9486703; doi:10.3389/fmicb.2022.986837)
Supplement: Supplementary file 1 [file Data_Sheet_1.docx]

**Supporting Information**

**Table S1. The microbial number of BI516 and LP-Onlly freeze-dried powder**

| Freeze-dried powder | Test Item | Microbial number | Test Method |
| --- | --- | --- | --- |
| BI516 | *Bifidobacterium lactis* | 3.2×10^11^ CFU/g | GB 4789.35-2016 |
| LP-Onlly | *Lactobacillus plantarum* | 1.6×10^11^ CFU/g | GB 4789.35-2016 |

**Table S2. Physiological and biochemical characteristics of LP-Onlly**

| Control | Glycerin | Erythritol | D-arabinose | L-arabinose | Ribose | D-xylose |
| --- | --- | --- | --- | --- | --- | --- |
| - | - | - | - | - | + | - |
| L-Xylose | Adonol | Methyl β-xylobioside | Galactose | Glucose | Fructose | Mannose |
| - | - | - | + | + | + | + |
| Sorbose | Rhamnopyranose | Dulcitol | Inositol | Mannitol | Sorbitol | [Methyl α-D-mannopyranoside](https://www.chemsrc.com/en/cas/617-04-9_1114050.html) |
| **-** | **-** | **-** | **-** | **+** | **+** | **+** |
| α-D-Methylglucoside | N-acetylglucosamine | Amygdalin | Arbutin | Esculin | Salicyl alcohol | Cellobiose |
| **-** | **+** | **+** | **+** | **+** | **+** | **+** |
| Maltose | Lactose | Melibiose | Sucrose | Trehalose | Inulin | Melezitose |
| **+** | **+** | **+** | **+** | **+** | **-** | **+** |
| Raffinose | Amylum | Glycogen | Xylitol | Gentiobiose | Turanose | D-Lyxopyranose |
| **+** | **-** | **-** | **-** | **+** | **+** | **-** |
| D-Tagatose | D- Fucose | L- Fucose | D-arabinitol | L-arabinitol | Glucoheptonate | 2-Keto-gluconate |
| **-** | **-** | **-** | **-** | **-** | **+** | **-** |
| 5-Keto-gluconate |  |  |  |  |  |  |
| **-** |  |  |  |  |  |  |

Note: “+” means positive reaction, “-” means negative reaction

**Table S3. Physiological and biochemical characteristics of BI516**

| Control | L-Arabinose | Ribose | D-Xylose | D-Xylose | Adol | Galactose |
| --- | --- | --- | --- | --- | --- | --- |
| **-** | **-** | **+** | **+** | **-** | **-** | **+** |
| Glucose | Fructose | Mannose | Sorbose | Rhamnose | Dulcitol | Inositol |
| **+** | **+** | **-** | **-** | **-** | **-** | **-** |
| Mannitol | Sorbitol | Methyl α-D-mannopyranoside | N-acetylglucosamine | Amygdalin | Esculin | Salicin |
| **-** | **+** | **-** | **-** | **-** | **+** | **+** |
| Cellobiose | Maltose | Lactose | Melibiose | saccharose | Trehalose dihydrate | Inulin |
| **+** | **+** | **+** | **+** | **+** | **-** | **-** |
| Melezitose | Raffinose | Amylum | Glycogen | Gentiobiose | Glucoheptonate |  |
| **-** | **+** | **-** | **-** | **+** | **-** |  |

Note: “+” means positive reaction, “-” means negative reaction

**Sequence S1. 16S rRNA gene sequence of BI516**

GGATGAACGCTGGCGGCGTGCTTAACACATGCAAGTCGAACGGGATCCCTGGCAGCTTGCTGTCGGGGTGAGAGTGGCGAACGGGTGAGTAATGCGTGACCAACCTGCCCTGTGCACCGGAATAGCTCCTGGAAACGGGTGGTAATACCGGATGCTCCGCTCCATCGCATGGTGGGGTGGGAAATGCTTTTGCGGCATGGGATGGGGTCGCGTCCTATCAGCTTGTTGGCGGGGTGATGGCCCACCAAGGCGTTGACGGGTAGCCGGCCTGAGAGGGTGACCGGCCACATTGGGACTGAGATACGGCCCAGACTCCTACGGGAGGCAGCAGTGGGGAATATTGCACAATGGGCGCAAGCCTGATGCAGCGACGCCGCGTGCGGGATGGAGGCCTTCGGGTTGTAAACCGCTTTTGTTCAAGGGCAAGGCACGGTTTCGGCCGTGTTGAGTGGATTGTTCGAATAAGCACCGGCTAACTACGTGCCAGCAGCCGCGGTAATACGTAGGGTGCGAGCGTTATCCGGATTTATTGGGCGTAAAGGGCTCGTAGGCGGTTCGTCGCGTCCGGTGTGAAAGTCCATCGCCTAACGGTGGATCTGCGCCGGGTACGGGCGGGCTGGAGTGCGGTAGGGGAGACTGGAATTCCCGGTGTAACGGTGGAATGTGTAGATATCGGGAAGAACACCAATGGCGAAGGCAGGTCTCTGGGCCGTCACTGACGCTGAGGAGCGAAAGCGTGGGGAGCGAACAGGAT

**Table S4. Clinical score of glioma mice**

| Behavioral and neurological symptoms in mice | Grade |
| --- | --- |
| active, strong and fast movements, normal exploratory behavior | 1 |
| active with some interruptions of activity, exploratory behavior | 2 |
| prolonged lack of activity, limited exploratory behavior upon external stimuli, intact muscle tonus | 3 |
| ataxia, no exploratory behavior, severely limited reaction to external stimuli, decreased muscle tone, hunched posture, ruffled fur, delayed righting reflex | 4 |
| no righting reflex, premortal stage | 5 |

**Table S5. Beam balance test score**

| Behavior of mice | score |
| --- | --- |
| Balances with steady posture | 0 |
| Grasps side of beam | 1 |
| Hugs beam and 1 limb falls down from beam | 2 |
| Hugs beam and 2 limbs fall down from beam, or spins on beam (>60 s) | 3 |
| Attempts to balance on beam but falls off (>40 s) | 4 |
| Attempts to balance on beam but falls off (>20 s) | 5 |
| Falls off; no attempt to balance or hang on to beam (<20 s) | 6 |

**Table S6. PCR primer sequences**

| Gene | Forword (5’-3’) | Reverse (3’-5’) |
| --- | --- | --- |
| PTEN | TGGATTCGACTTAGACTTGACCT | GCGGTGTCATAATGTCTCTCAG |
| Survivin | GAGGCTGGCTTCATCCACTG | ATGCTCCTCTATCGGGTTGTC |
| Spp1 | CACTCCAATCGTCCCTACAGT | CTGGAAACTCCTAGACTTTGACC |
| N-cadherin | AGCGCAGTCTTACCGAAGG | TCGCTGCTTTCATACTGAACTTT |
| E-cadherin | CAGGTCTCCTCATGGCTTTGC | CTTCCGAAAAGAAGGCTGTCC |
| GAPDH | AGCGAGACCCCACTAACATC | GGTTCACACCCATCACAAAC |

**
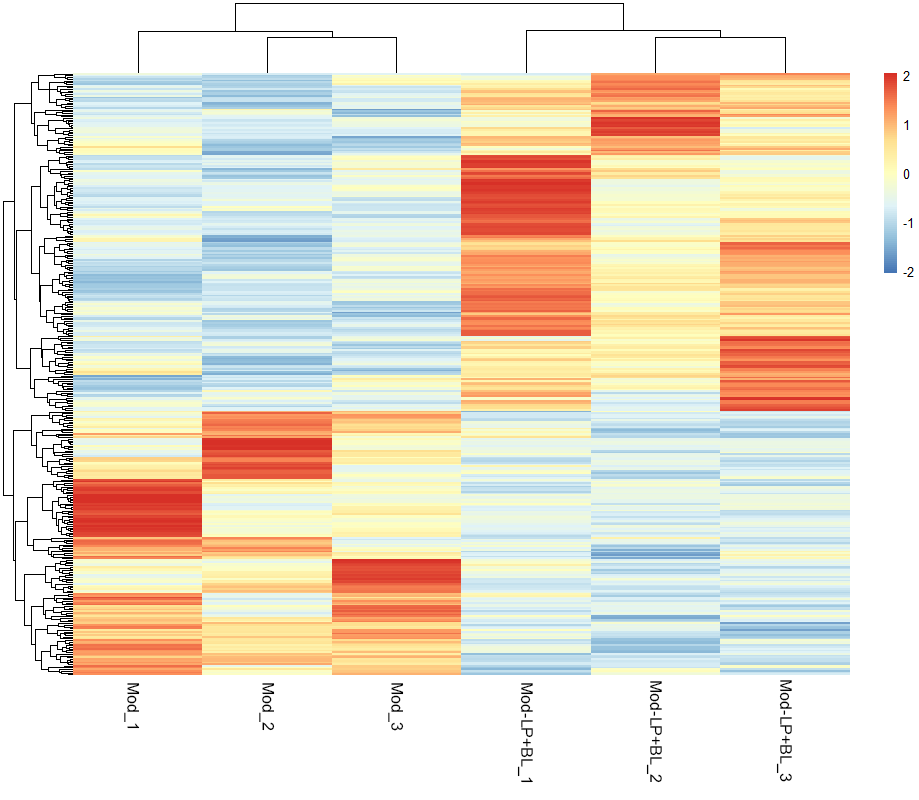
**

**Figure S1. The expression profiles of DEGs between different groups.** Mod, Model. LP, *Lactobacillus plantarum*. BL, *Bifidobacterium lactis*. n = 3 for each group.

**
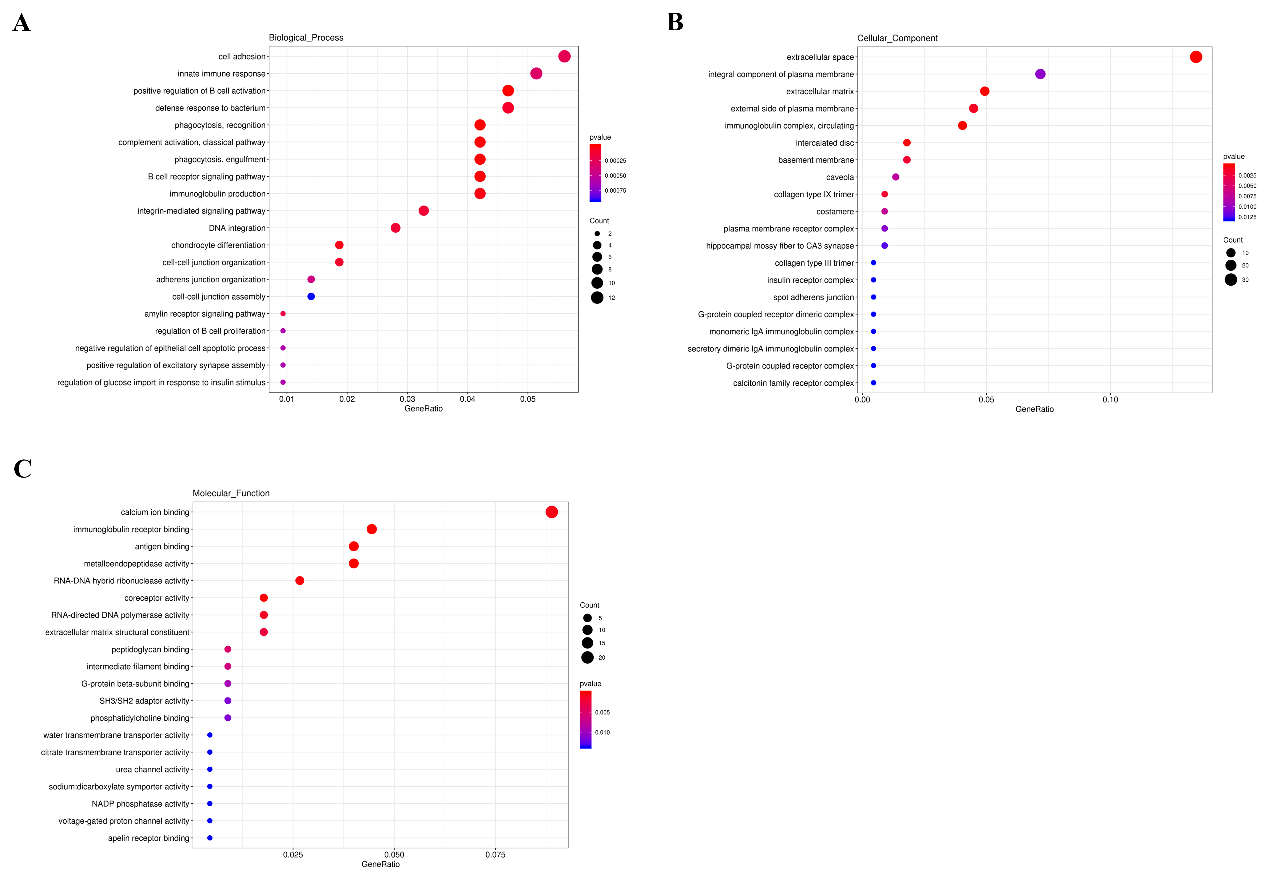
Figure S2. GO enrichment analysis of the DEGs.** (A) Biological process, (B) cellular component and (C) molecular function items of the DEGs.

**
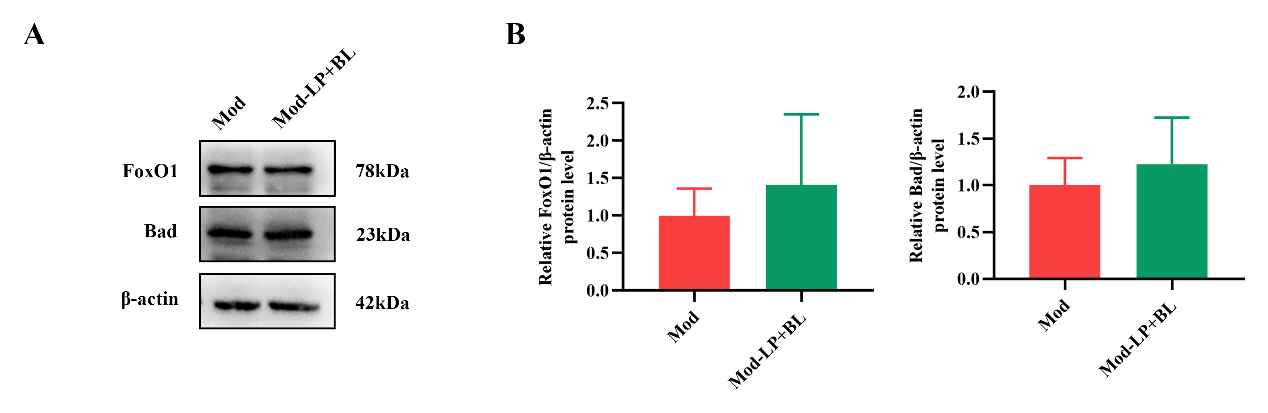
**

**Figure S3. The protein expression of Bad and FoxO1 in glioma tissues of mice.** (A) Representative immunoblots and (B) quantitative analysis of Bad and FoxO1 in glioma tissues. n = 6. Two-way *ANOVA*. Mod, Model. LP, *Lactobacillus plantarum*. BL, *Bifidobacterium lactis*.

**
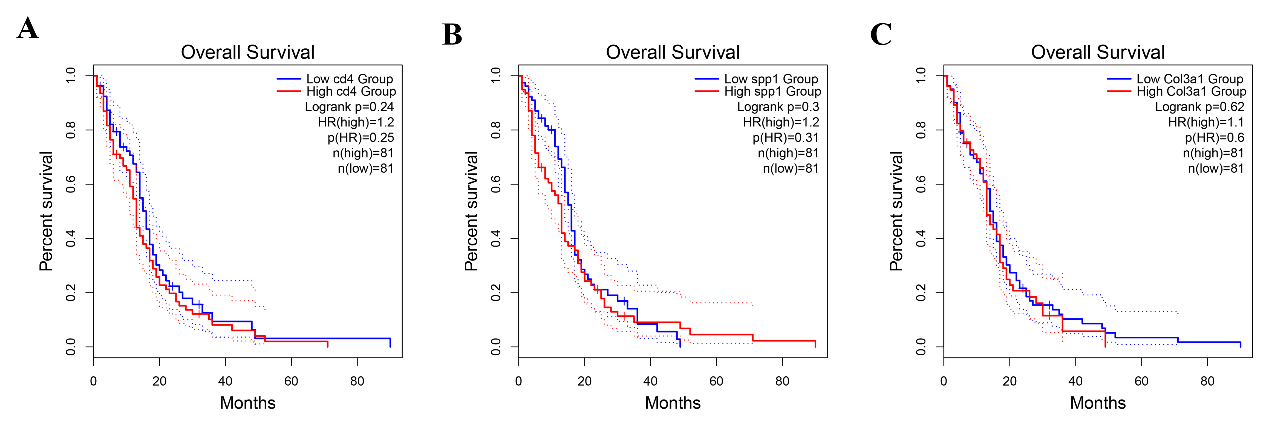
**

**Figure S4.** **Survival analysis for hub genes.** Kaplan-Meier overall survival curves of glioma patients according to expression levels of (A) *CD4*, (B) *Spp1* and (C) *Col3a1*.

**
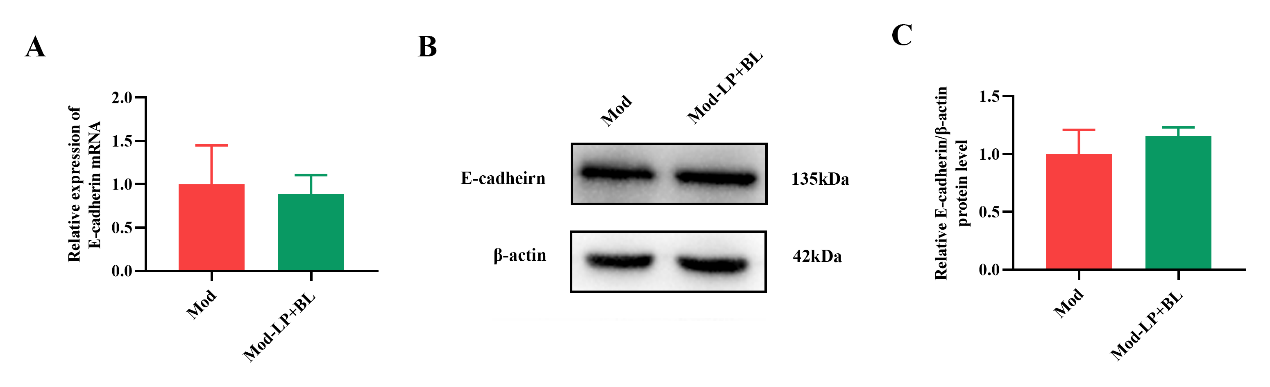
**

**Figure S5. The mRNA and protein expression of E-cadherin in glioma tissues.** (A) mRNA expression of E-cadherin in glioma tissues. (B) Representative immunoblots and (C) quantitative analysis of E-cadherin in glioma tissues. n = 6. Two-way *ANOVA*. Mod, Model. LP, *Lactobacillus plantarum*. BL, *Bifidobacterium lactis*. ^*^*P* < 0.05, ^**^*P* < 0.01, ^***^*P* < 0.001.


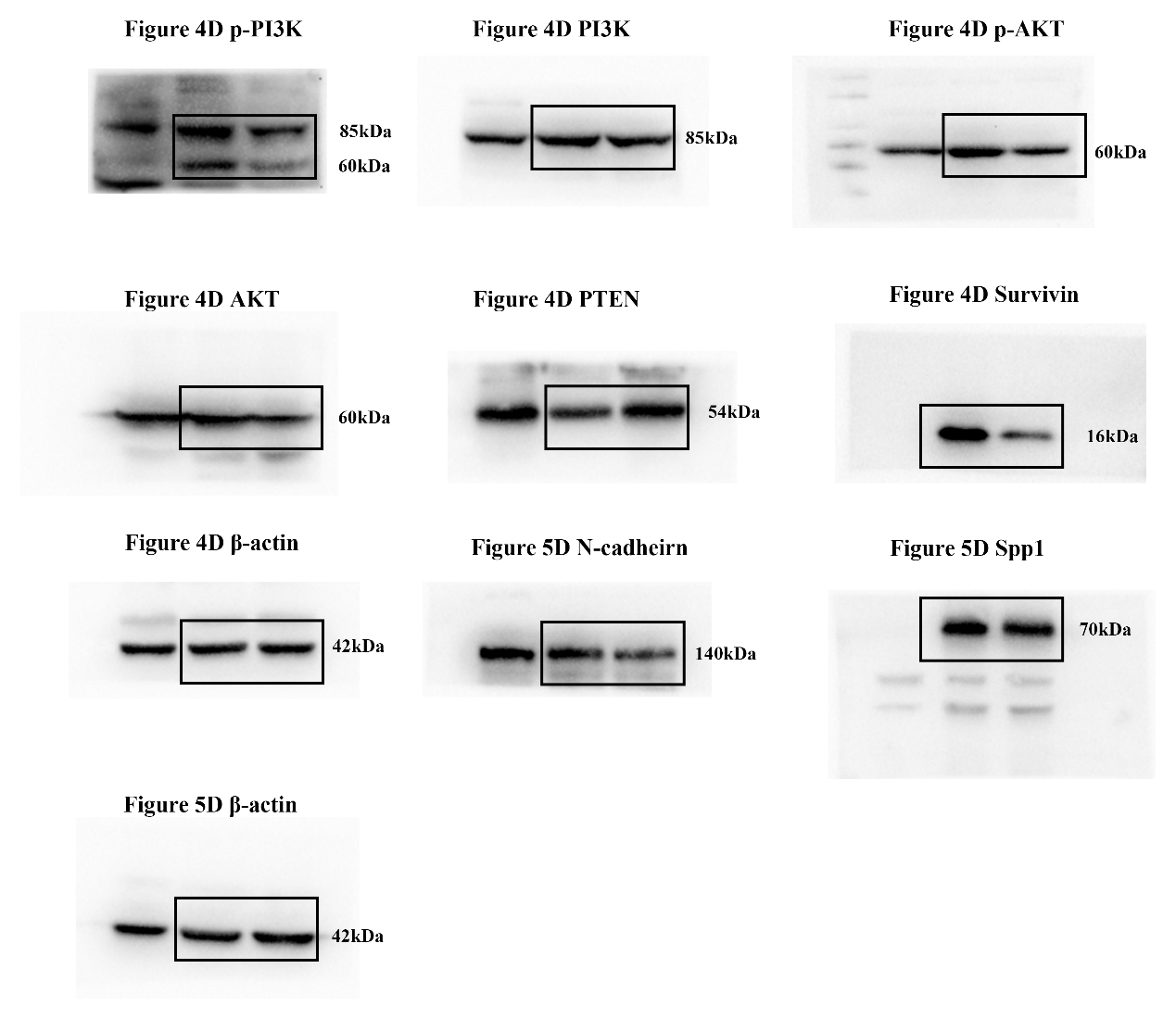


**Figure S6. Original images of Western blot.** The bands from left to right respectively represent Sham, Mod and Mod-LP+BL group.


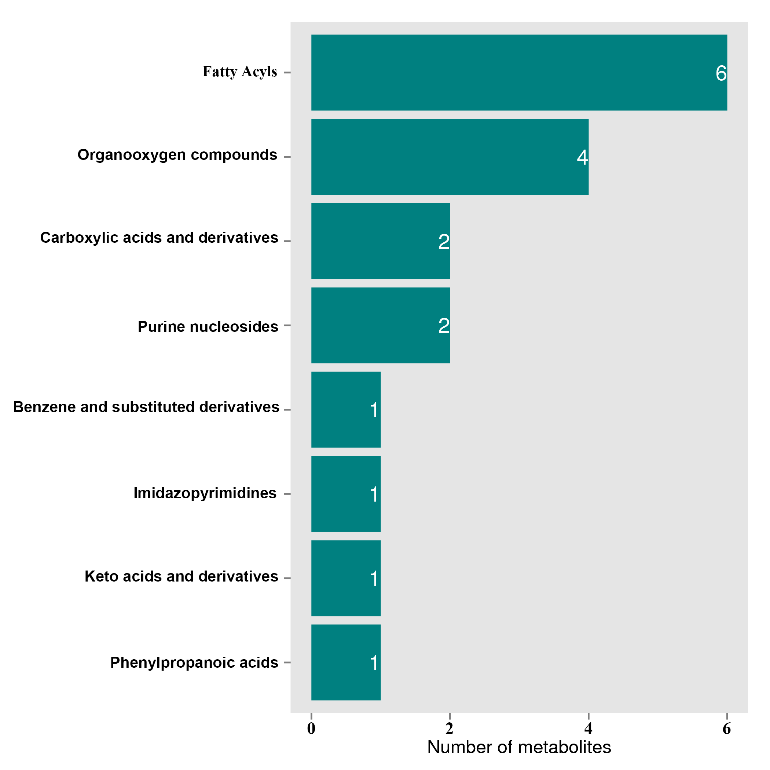


**Figure S7. HMDB classification map of differential metabolites.**


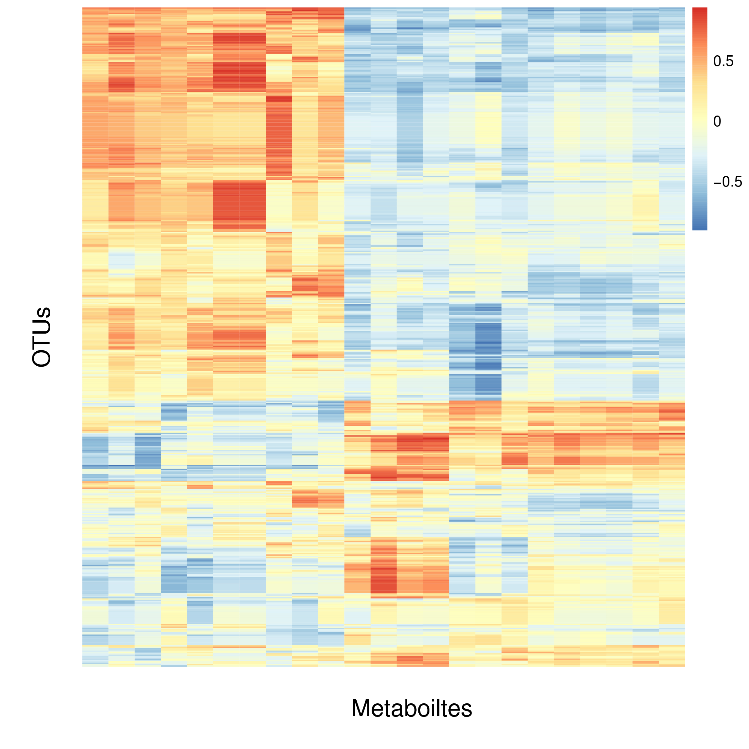


**Figure S8. Correlation analysis between OTU and all differential fecal metabolites.** Spearman’s rank correlation. n = 5-6.
